# Micromechanical Model for Predicting the Tensile Properties of *Guadua angustifolia* Fibers Polypropylene-Based Composites

**DOI:** 10.3390/polym14132627

**Published:** 2022-06-28

**Authors:** Jorge I. Fajardo, Josep Costa, Luis J. Cruz, César A. Paltán, Jonnathan D. Santos

**Affiliations:** 1 New Materials and Transformation Processes Research Group GiMaT, Universidad Politécnica Salesiana, Cuenca 010102, Ecuador; cpaltan@ups.edu.ec (C.A.P.); jsantos@ups.edu.ec (J.D.S.); 2 Analysis and Advanced Materials for Structural Design AMADE, Polytechnic School, University of Girona, Campus Montilivi s/n, E-17003 Girona, Spain; josep.costa@udg.edu; 3 New Materials Research Group GINUMA, Universidad Pontificia Bolivariana, Circular Primera y Bolivariana, Medellín 56006, Colombia; luis.cruz@upb.edu.co

**Keywords:** natural fiber, biobased composite, micro-mechanics, polymer-matrix composites (PMCs), X-ray computed tomography

## Abstract

In this paper, the one-dimensional tensile behavior of *Guadua angustifolia* Kunth fibre/polypropylene (PP+GAK_S_) composites is modeled. The classical model of Kelly–Tyson and its Bowyer–Bader’s solution is not able to reproduce the entire stress–strain curve of the composite. An integral (In-Built) micromechanical model proposed by Isitman and Aykol, initially for synthetic fiber-reinforced composites, was applied to predict micromechanical parameters in short natural fiber composites. The proposed method integrates both the information of the experimental stress-strain curves and the morphology of the fiber bundles within the composite to estimate the interfacial shear strength (IFSS), fiber orientation efficiency factor $\eta_{\mathrm{FOD}}$, fiber length efficiency factor $\eta_{\mathrm{FLD}}$ and critical fiber length $l_{c}$. It was possible to reproduce the stress-strain curves of the PP+GAK_S_ composite with low residual standard deviation. A methodology was applied using X-ray microtomography and digital image processing techniques for the precise extraction of the micromechanical parameters involved in the model. The results showed good agreement with the experimental data.

## 1. Introduction

The traditional continuous synthetic fiber reinforced polymer (FRP) has been exposed superior properties, especially their high strength-to-weight ratio and applications at several fields such as F-1 cars, sport accessories, aeronautics and aerospace, and ship construction [1,2,3]. Nonetheless, the entire design procedure for these kind of materials is a non-straightforward task, taking into account the fragile behavior without consistent plastic deformation for high anisotropic materials. This mechanical response has been modeled by using a bilinear stress–strain curve with a linear elastic zone and a softening region [4,5]. Hence, a complex stress field in the material is experienced under a general loading case (flexural, tensile, and compression), which is compared with the corresponding yielding property through testing. The Tsai–Wu theory in 1970 was one of the pioneer models to propose a failure criterion based on the macroscopic yielding of anisotropic metals predicting the yield limit [6]. The development of failure criteria based on yielding determination derived from Hashin (1980), Chang-Lessard (1991), Puck (1998), Cuntze (2004), Azizi (2012), and Daniel (2015), which analyzed composites under the mixed-mode case [7,8,9,10,11,12,13]. 

Taking into account that in the industrial field, most products are made with multidirectional (MD) composite laminates. Most designers used conservative design philosophies of unidirectional (UD) composite lamina failure by means of the ultimate calculated strength from experimental data [14]. The last is considered as the yield strength of the entire composite. This is resulting in MD FRP composite parts that can sustain up to 10 times more loading without experience matrix and interface failure [14,15,16]. To deal with this issue, a recent work by Koloor et al. [7] proposed a novel concept based on the damage dissipation energy (DDE) by means of the multiple softening processes under several mesoscale failure modes. This new energetic concept can increase the yield limit between 30–50% of the maximum loading capacity of the entire structure, which is an increase of 20–30% in relation to the tradition design basis.

On the other hand, in the framework of the short natural fiber reinforced composites (SNFC), despite the high development of this kind of materials their massive applications have been limited to markets that require low costs, high production rates and moderate mechanical properties [17,18,19]. One of the aspects that has limited the potential uses of these materials are the difficulty of predicting their mechanical properties on a final product and the determination of an adequate compendium of processing conditions avoiding time consuming explorations. The first is due to the complex physical and chemical interactions between their constituents, which can experience high variability based on the processing conditions. The exploration of the tensile micromechanical models for SNFC reported by Kelly and Tyson in 1965 [20] is one of the first contribution, this work concluded that existed a linear relation between the ultimate tensile strength and the quantity of the tungsten and molybdenum fibers into a cooper matrix. Chronologically, the misalignment in short fibers was studied by Bowyer and Bader [21]; the authors proposed a model to estimate the fiber-matrix bonding strength derived from the stress-strain test of the composite. Short glass fiber reinforced polyamide 6.6 and polypropylene (PP), respectively, the batch of specimens were tested to validate the model. Results concluded that the tensile performance increased by modifying processing parameters as flow. The two previous models were proposed to deal with traditional composite materials; however, they have been implemented to analysis SNFC. Espinach et al. in 2014 [22] explored the tensile response of a biocomposite, alpha-grass 5–30 wt%. reinforced starch-based polymer matrix. The micromechanical models predicted the tensile modulus according to several equations such as Hirsch, Tsai–Pagano, Halpin–Tsai, and a modified role of mixtures (m-ROM). Numerical results were in good agreement with experimental ones. Moreover, the biocomposite at 30 wt%. exposed similar tensile performance than short glass fiber (SGF) reinforced PP.

The numerical tensile micromechanical models have been used for exploration of potential natural source reinforcing composite. Serrano et al. [23] analyzed the incorporation of old newspaper fibers (ONF) reinforcing PP, through the Cox–Krenchel, Tsai–Pagnano, Halpin–Tsai and Hirsch models. The last was useful to calculate the intrinsic Young’s modulus from the experimental tensile test, which might be impossible to measure through an experimentation campaign. All numerical models reported similar trends. The next phase of the implementation of the micromechanical tensile models was reported by Serrano et al. [24]; the paper evaluated the design process of a single-cell centrifugal water pump according to m-ROM. Stiffness of the ONF at 30 wt%. reinforcing PP was able to reach 70% stiffness of SGF reinforced PP composite. Continuing with the exploration of potential natural source of fiber reinforced composite, Reixach et al. [25] analyzed the tensile behavior of PP reinforced with orange pruning-short fibers by means of both tensile testing campaign and numerical prediction according to m-ROM. They concluded that the biocomposite at 50 wt%. is mechanically comparable with SGF at 20 wt%. reinforced PP. Granda et al. [26] experimentally studied the tensile behavior of Leucaena collinsii reinforced PP as well as the m-ROM and Cox–Krenchel models were used to predict the mechanical tensile performance. Results denoted that fiber could increase the stiffness around 400% in relation to the neat polymer; good agreement between results and fiber diameters was not a relevant factor for intrinsic stiffness of the fiber.

Notta-Cuvier et al. [27] proposed an adaptation of the Bowyer–Bader model to compute the one-dimensional stress state for monotonic tensile test of each fiber, short-flax fiber reinforced PP exposed good agreement between predicted and experimental results. The proposed model revealed that the fiber length and the interfacial shear strength (by using shear lag model) were key parameters, as was expected. In a similar work reported by Anderson et al. [28], who proposed a modified Bowyer–Bader model to predict the tensile behavior of a short fiber reinforced composite based on the stress-strain curve. The numerical evaluation of the short-flax fiber reinforced starch acetate composite exposed that the apparent interfacial shear strength was found to be depended on the fiber loading. Xiong et al. [29] studied the tensile behavior of short-flax fiber reinforced PP by proposing a modified shear lag model based on a cohesive fiber/matrix interface and a finite element (FE) simulation using cohesive law for comparison. Simulated and predicted results exposed good agreement with experimental results; afterwards, the proposed model had similar trend with the Halpin–Tsai model. In a recent work reported by Pantano et al. [30], a numerical study was carried out by considering the effects of the volume fraction, average length fiber, curvature and distribution of the fiber orientation on the tensile behavior of short sisal fibers at 35 wt%. reinforcing green thermosetting epoxy. The micromechanical numerical model was implemented on a representative volume element (RVE), experimental results had good agreement with the predict ones. 

According to the literature reviewed by the authors, most micromechanical numerical models were proposed based on a certain modification of shear lag; Kelly–Tyson, Bowyer–Bader, and Halpin–Tsai, mainly. Some works were focused on comparing the accuracy of the model for the determination of the elastic modulus with experimental results. This accuracy for modelling and predicting SNFC tensile behavior can be affected by the fiber orientation, volume fraction, and fiber length, which may be totally different depending on location in manufacturing parts specially for injection molding technique [31,32]. Hence, the present work determines the one-dimensional tensile behavior of *Guadua angustifolia* Kunth fibre/polypropylene (PP+GAK_S_) composites by means of the In-Built micromechanical model for predicting the entire stress-strain curve from a monotonic tensile load until failure. The micromechanical parameters needed for the model such as fiber diameter, fiber length, and fiber orientation were captured by means of microtomography and digital image processing.

## 2. Micromechanical Models

### 2.1. The Bowyer–Bader Discrete Solutions

Literature reports a variety of studies that estimate the IFSS and the orientation efficiency factor ($\eta_{\mathrm{FOD}}$) with reasonable validity in natural composite materials based on the Bowyer–Bader methodology [20,21], (Equations (1) and (2)).

(1) $$\sigma_{c}\left(\varepsilon \right)=\eta_{\mathrm{FOD}}\left[\overset{l_{i}<l_{c}}{\underset{i}{\sum }}\left(\frac{\tau l_{i}^{F}V_{i}^{F}}{d}\right)+\overset{l_{i}>l_{c}}{\underset{j}{\sum }}E_{f}\varepsilon V_{j}^{F}\left(1-\frac{l_{c}}{2l_{j}^{F}}\right)\right]+\left(1-V^{F}\right)E_{m}\varepsilon$$

(2) $$l_{c}=\frac{E_{f}\varepsilon d}{2\tau }$$

where $\sigma_{c}$ represents the tensile stress of the composite, $\eta_{\mathrm{FOD}}$ is the orientation efficiency factor, τ is the interfacial shear strength, $l_{i}$ and $l_{j}$ represent the nth-subcritical and nth-supercritical fiber length respectively, $V_{i}$ and $V_{j}$ represent the volumetric fraction of sub and supercritical fibers, d is the average diameter of fiber, $l_{c}$ is the critical fiber length at composite strain, defined by Equation (2), $E_{f}$ is the fiber young modulus, and ε is the composite strain. The Bowyer–Bader methodology was used originally to evaluate IFSS and $\eta_{\mathrm{FOD}}$ from experimental stress-strain curves at two arbitrary points of strain, assuming a linear elastic matrix response. This method has been applied to evaluate micromechanical parameters of SNFC such as the tensile stress and fiber orientation in kenaf fiber/starch-grafted polypropylene composites [33], the tensile strength in nonwoven kenaf fiber/epoxy composites [34], the tensile strength in cellulose fiber reinforced nylon 6 or nylon 66 composites [35], the apparent IFSS in short-flax-fiber/starch acetate composites by a modified Bowyer–Bader method that includes a shear lag model allowing for an elastic-perfectly plastic stress transfer [28], and the IFSS$,\;\eta_{\mathrm{FOD}}$ and mean equivalent intrinsic tensile strength of old newspaper fiber/polypropylene composite [18].

### 2.2. Integral (In-Built) Model

Other analytical models have been developed taking into account the whole experimental stress-strain curve of the composite instead of two points. Isitman et al. [36,37] demonstrated that the discrete two-term model proposed by Kelly–Tyson could be treated by a continuous integral micromechanical analysis “In-Built”. Under this approach, it is possible to estimate the IFSS and $\eta_{\mathrm{FOD}}$ more accurately than the aforementioned models. By means of FLD it is possible to take into account the fiber breakage phenomena during extrusion and injection process of SNFC at different fiber contents. It is assumed a stochastic fiber length distribution that can be well modeled by the Weibull distribution function (Equation (3)).

(3) $$P_{l}=Knl^{n-1}e^{-Kl^{n}}dl$$

where $P_{l}$ is the probability density of fibers with a length between l and l+dl interval; K and n represent the scale and shape parameters of Weibull distribution. Discrete Equation (1) can be converted into integrals over entire fiber lengths by Equation (4):

(4) $$\sigma_{c}\left(\varepsilon \right)=\eta_{\mathrm{FOD}}Kn\left[\int_{0}^{l_{c}}\frac{\tau }{d}l^{n}e^{-Kl^{n}}dl+\int_{l_{c}}^{\infty }E_{f}\varepsilon \left(1-\frac{l_{c}}{2l}\right)l^{n-1}e^{-Kl^{n}}dl\right]V_{f}+\left(1-V_{f}\right)\sigma_{m}\left(\varepsilon \right)$$

from equation (4) it can be seen the non-linear function of the contribution of matrix to the composite strength.

On the other hand, the modified rule of mixtures (m-ROM) includes two efficiency factors $\eta_{\mathrm{FOD}}$ and $\eta_{\mathrm{FLD}}$ for orientation and fiber length respectively, Equation (5):

(5) $$\sigma_{c}=\eta_{\mathrm{FOD}}\eta_{\mathrm{FLD}}\sigma_{f}V_{f}+\sigma_{m}\left(1-V_{f}\right)$$

As proposed by Isitman et al. [36,37], it is possible to define B as:

(6) $$B=\eta_{\mathrm{FLD}}\sigma_{f}$$

By comparing Equations (4) and (5), then B can be written as:

(7) $$B=Kn\left[\int_{0}^{l_{c}}\frac{\tau }{d}l^{n}e^{-Kl^{n}}dl+\int_{l_{c}}^{\infty }E_{f}\varepsilon \left(1-\frac{l_{c}}{2l}\right)l^{n-1}e^{-Kl^{n}}dl\right]$$

Solving Equation (7)

(8) $$B=Kn\left[\left(\frac{\tau }{d}\right){\frac{-nle^{-Kl^{n}}-l{\left(Kl^{n}\right)}^{-\frac{1}{n}}\;\Gamma \left(\frac{1}{n},\;Kl^{n}\right)}{Kn^{2}}|}_{0}^{l_{c}}-{\frac{E_{f}\varepsilon e^{-Kl^{n}}}{Kn}|}_{l_{c}}^{\infty }-{\frac{E_{f}\varepsilon \Gamma \left(1-\frac{1}{n},\;Kl^{n}\right)}{2nK^{1-\frac{1}{n}}}|}_{l_{c}}^{\infty }\right]\;$$

Since $\underset{l\to \infty }{\lim}\Gamma \left(\frac{1}{n},Kl^{n}\right)=0$, and $\underset{l\to \infty }{\lim}e^{-Kl^{n}}=0$.

B can be expressed as:

(9) B=(τd)[−lce−Klcn− Γ(1n, Klcn)−Γ(1n)nK1n]+Efεe−Klcn−Efεlc2K1nΓ(1−1n, Klcn)  

From equilibrium in the interface $\frac{\tau }{d}=\frac{E_{f}\varepsilon }{2l_{c}}$ and substituting Equation (9) in Equation (6), it is possible to define a unified parameter that takes the FLD into account. The FLD efficiency parameter can be computed as:

(10) ηFLD(ε)=12[e−Klcn− Γ(1n, Klcn)−Γ(1n)lcnK1n−lcK1nΓ(1−1n, Klcn)]  

It is clear that $\eta_{\mathrm{FLD}}\;$is a function of applied strain and by substituting Equation (10) into Equation (5), the m-ROM equation can be written as:

(11) σc=12[e−Klcn− Γ(1n, Klcn)−Γ(1n)lcnK1n−lcK1nΓ(1−1n, Klcn)]ηFODσfVf+σm(1−Vf) 

Now it is possible to model the composite tensile strength as a function of strain at different fiber contents with a FLD defined by *K* and *n* parameters.

There are more complex models for short fiber reinforced composites (SFCs), including matrix viscoelastic–viscoplastic behavior [38], dynamic behavior [39], and progressive fiber/matrix debonding [31] applications that require a complex characterization process and extensive numerical analysis [27,31,39], turning those models as non-industry-friendly.

## 3. Materials and Methods

### 3.1. Guadua, Polymer and Coupling Agent

Bamboo fibers (specie *Guadua angustifolia* Kunth) with length 0.5 ± 0.1 mm, diameter 98.15 ± 0.1 μm (cross section was determined by optical microscopy as the average of at least 5 measurements) and density of 1.27 ± 0.0028 g/cm^3^ (obtained by the authors through a helium pycnometry analysis according to ASTM D5550-14), from the coastal region of Ecuador were used as a reinforcing. The fibers were isolated from the culm by means a thermomechanical extraction process, namely, steam explosion. The tensile strength and Young’s modulus of the GAK_S_ fiber bundles were evaluated according to ASTM D3822-07, using a texture analyzer, TA.XT plus supplied by Stable Micro Systems, cross-head speed of 1 mm/ min, under standard environmental conditions according to ASTM 1775. The values of tensile strength and Young’s modulus of GAK_S_ fibers were 452.52 ±133 MPa and 21.13 ±6 GPa, respectively. 

Polypropylene homopolymer (PP) provided by Total Refining & Chemicals, density of 0.905 g/cm^3^, tensile strength of 25.82 MPa, and Young’s modulus 1.21 GPa, was used as polymer matrix. Polypropylene grafted with maleic anhydride (MAPP) supplied by Sigma Aldrich, average molecular weight Mw ~ 9100, and density of 0.934 g/mL at 25 °C, was used as a coupling agent.

### 3.2. Extrusion of the Composite

Composite materials at different formulations of GAK_S_ (30 to 40 wt%) and MAPP (4 to 8 wt%) were manufactured in a twin-screw extruder (Clextral BC 21, Firminy, France) equipped with two automatic volumetric feeders. The extruded material was cooled in a water bath and subsequently pelleted (length ≤ 4 mm). To reduce the moisture content, the pellets were dried in an oven at 80 °C for at least 12 h and then stored in vacuum sealed bags.

### 3.3. Injection Molding

Tensile test specimens according to ISO 527 Type 1B standard (150 × 4 × 10 mm^3^) were injected on a DK-Codim injection machine (NGH 50/100, Gonesse, France). The injection temperature was 190 °C. Composite materials were made in different formulations of GAKS (30 to 40 wt%) and MAPP (4 to 8 wt%).

### 3.4. Mechanical Characterization

**Figure 1:**
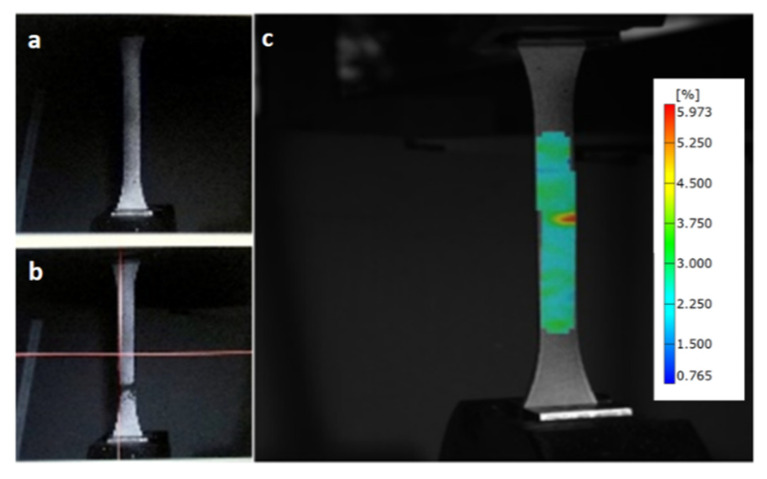
Tensile test specimen coated with randomly speckles: (**a**) before testing, (**b**) after testing, and (**c**) detail of the post-processing (strain field).

The specimens were previously conditioned according to ASTM D618-13, procedure A, and condition 40/23/50. Quasi-static tensile tests were carried out according to ASTM 638 using an Instron 33R 4204 testing machine equipped with 20 kN loading cell and operating at rate of 5 mm min^−1^. Axial displacements were recorded using a Digital Image Correlation (DIC) system. Images were taken using two cameras equipped with lenses of 35 mm focal length and controlled by the ARAMIS system. The sets of digital images were taken every second during straining. These data served as input to calculate the displacement field using the DIC method [40]. The least square criteria based on the sum of the least square minimums of the facet terms was applied. The correlation factor that tends to a minimum value or to zero when the deformed position of the facet is located [41]. The algorithm consists of moving the facet around its initial position until the function that defines the correlation factor is minimized. For each new position, the matrix that defines the new facet is subtracted from its initial position, and the terms of the resultant matrix are squared and summed to obtain the correlation factor. The DIC system tracks the movement of a random pattern painted on the specimen surface (speckles of black color on a white background) [42]. The painted pattern was random, isotropic, and high contrast (Figure 1a,b); the region of interest used by the optical system was 15 mm × 50 mm. ARAMIS professional software was employed for the post-processing (Figure 1c). The results were obtained from the average of at least 10 samples.

### 3.5. Microtomography X (μ-CT)

For the analysis of the microstructure of SNFC, a non-destructive technique was applied by means of X (μ-CT) [43]. With this technique, it was possible to recreate images of the internal structure of the composite material and evaluate their microstructure (fiber length, diameter and orientation distribution). The images were created from the central area of the PP+30GAK_S_+4M and PP+40GAK_S_+8M specimens. The images were acquired with a resolution of 6.5 μm using a voltage of 45 kV and current of 160 μA, by means a Novadep micro-tomograph according to the method described by Paltán in [44]. The reconstructed three-dimensional images were filtered and subsequently thresholded to identify fibers, pores and matrix. The aspect ratio, diameter, and length of the fibers were determined from 100,000 samples of the cross section by the method developed by Miettinen et al. [43]. The orientation efficiency factor was determined by the Krenchel model:

(12) $$\eta_{o}=\underset{n}{\sum }a_{n}cos^{4}\alpha_{n}$$

where $\alpha_{n}$ is the angle between the main axis of the nth fiber and the flow direction [38,39,40,41,42,43,45]. The distribution of fiber lengths, the distribution of diameters and the aspect ratio were evaluated. This method makes it possible to calculate the cross-sectional dimensions and length of non-circular and non-straight fibers by means estimations of local orientation of the fibers allowing the extraction of cross-sectional slices. The fiber length is estimated using the constrained path-opening method as in [38,39,40,41,42,43,45,46], given its ability to follow irregular paths and generate the regular, longer path that passes through each fiber. The results were expressed as distributions weighted by fiber volume.

### 3.6. Interfacial Shear Strength

Interactions between natural fibers and thermoplastic matrices are complex due to the physical interactions (entanglement, friction and Van der Waals forces) and chemical interactions (covalent and hydrogen bonds) [14]. This type of interaction works together to increase the capacity of load transfer between the fibers and matrices, improving their mechanical properties [47]. The most commonly used parameter to evaluate the fiber-matrix adhesion is the interface shear strength (IFSS). The IFSS values reported by the literature show significant differences between them and great variability for similar composites, depending on the preparation and the characterization technique. For those reasons, an integral mathematical modeling, proposed by Isitman and Aykol [36,37,48] was adjusted for use with natural fibers. The values of $E_{f}$, $E_{m}$, $\sigma_{m}$, $V_{f}$,ε, d, $\eta_{\mathrm{FOD}}$, and the fiber length distribution, represented by K and n parameters were entered in the model. From the model and by minimizing the error between the experimental data and the simulations, the IFSS (τ) was obtained.

## 4. Results and Discussion

### 4.1. Tensile Tests of Composite

**Figure 2:**
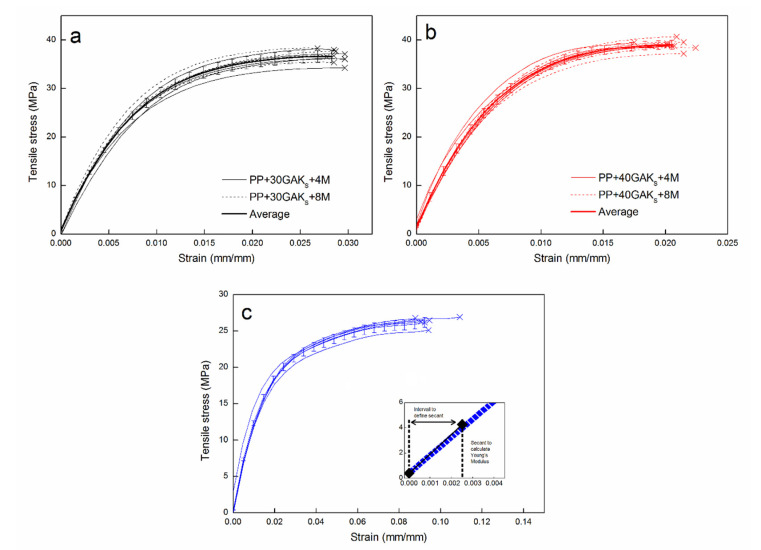
Experimental engineering stress-strain curves, (**a**) PP+30GAKs, (**b**) PP+40GAKs, and (**c**) PP without reinforcement with detail of the secant for evaluation of the modulus.

Experimental engineering tensile stress-strain curves of composites PP+30GAK_S_, PP+40GAK_S_, and raw PP matrix are shown in Figure 2a,b. As can be seen, the formulated composites show both brittle behavior and increased tensile strength compared to the unreinforced PP, Figure 2c. This effect is directly related to load transfer from PP matrix to the stiffer GAK_S_ fibers. The Young’s modulus was determined by the secant gradient in the range of the best linear fit of the experimental stress-strain curve (detail Figure 2c). Table 1, summarizes the experimental results for the formulated composites.

### 4.2. Microstructural Characterization by Using X (μ-CT)

**Figure 3:**
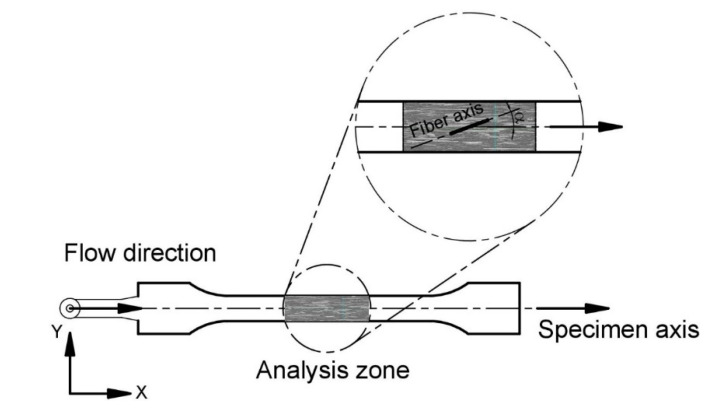
Scanning zone of PP+30 GAKs by microtomography X (μ-CT).

The microstructure of the composite materials was characterized for the compositions PP+30GAK_S_+4M and PP+40GAK_S_+8M. Geometric and microstructural properties were evaluated by X (μ-CT) from the central zone of the specimens (fully developed flow zone). Figure 3, shows the geometry of the specimen, the analysis zone, and the 3D reconstruction of the composite PP+30GAK_S_+4M. It was observed that most of the fibers are oriented in the flow direction. On the contrary, a very low orientation was observed in the out-of-plane direction (i.e., with respect to the thickness of the specimen). This planar orientation is typical for injected SNFC [49,50,51]. In order to evaluate the fiber orientation with respect to the flow direction (X axis), the inclination angle (α) and, subsequently, the orientation efficiency factor was calculated by the Krenchel model, Equation (10).

**Figure 4:**
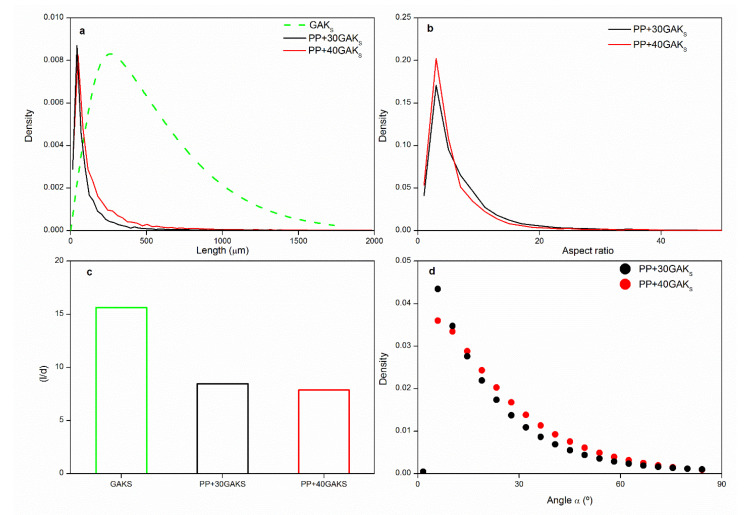
Micromechanical parameters: (**a**) fiber length distributions, (**b**) aspect ratio distribution of, (**c**) variation of the aspect ratio, and (**d**) fiber orientation distributions.

During the composite manufacturing process by extrusion and injection, the fibers undergo changes in their morphology expressed in the reduction of their length and diameter. The length distribution, diameter, aspect ratio, and fiber orientation are fundamental micromechanical parameters affecting the tensile properties of SNFC, therefore, an evaluation of the changes occurring during their processing is essential [18,23,49,50,52,53,54]. The micromechanical parameters were computed from tomographic images of the selected Figure 4. 

**Figure 5:**
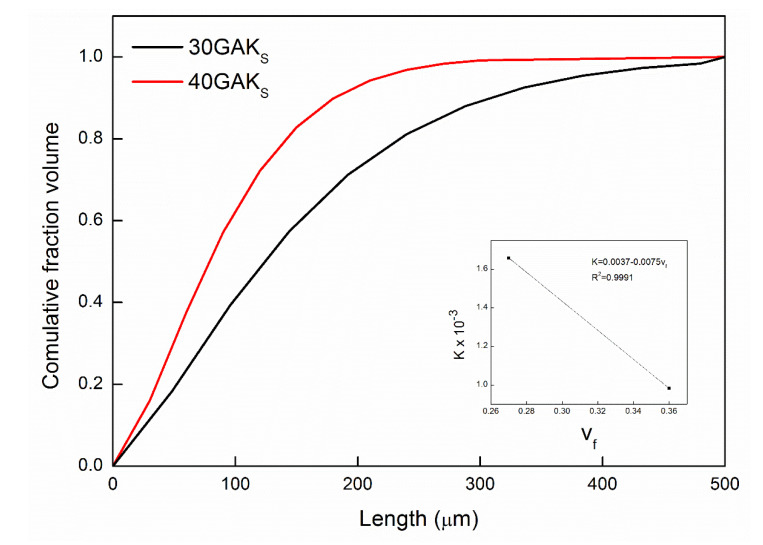
Variation of FLD in the PP+GAK_S_ composite with the fiber content.

Figure 4a, shows an important reduction of fiber length during the processing. The fiber length distribution has been shifted towards the shorter fibers. The average fiber length before processing was ~500 μm and after processing (extrusion + injection) it have become 241.86 μm and 165.26 μm in the PP+30GAK_S_+4M and PP+40GAK_S_+8M, respectively (Figure 4a). The obtained results showed similar trends as those obtained by Serrano et al. [18], Vallejos et al. [55], and López et al. [56] in injection-molded SNFC. The aspect ratio of the fiber bundles was reduced by approximately half, due to the size reduction (Figure 4b). The average fiber diameter before processing was found to be 33 μm, and it was reduced to 28.57 μm and 21.01 μm for the PP+30GAK_S_+4M and PP+40GAK_S_+8M, respectively. It was observed that the fiber content has greater influence on the length reduction rather than in the diameter reduction. This effect was attributed to the increase in shear flow and the fiber-fiber interactions which generate the fragmentation of slender fibers [50,51,53,54,55,56,57]. Despite this effect, the average aspect ratio showed similar values in both compositions after processing (Figure 4c). Joffre et al. [58] reported similar trends analyzing by X (μ-CT) the fiber length degradation of spruce reinforcing PLA, processed by extrusion+injection. Analyzing the distribution of fiber orientation, all sections showed a preferential orientation in the flow direction (α ~ 0°). Figure 5d presents the weighted and averaged orientation distributions through the thickness of the specimens, to obtain representative fiber orientation distributions.

Figure 5 shows the cumulative fiber length distribution function at different fiber bundle contents (30GAKs and 40GAKs). Weibull distribution function showed the best fitting with the experimental data. The distribution was shifted towards the shorter fibers by increasing the fiber content. The scale parameter (K) decreases while the fiber content increases (detail Figure 5).

### 4.3. Interfacial Shear Strength

The experimental data for modeling tensile behavior using the In-Built model are summarized in Table 2. Some parameters were obtained experimentally (Young’s moduli of the fiber and matrix, fiber diameter and ultimate strain). On the other hand, the scale and shape factors were obtained by means of a code programmed in Matlab R2018b from the data obtained by X-ray microtomography.

**Figure 6:**
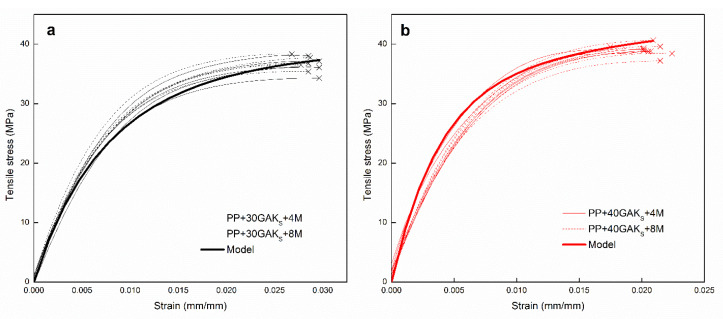
Tensile experimental (dashed lines) and theoretical (solid lines) stress-strain curves: (**a**) PP+30GAKs+4M and (**b**) PP+40GAKs+8M.

The In-Built model was applied to estimate the IFSS of PP+GAK_S_ composites at 30 and 40 wt%. A least squares optimization algorithm with heuristic minimization was used for a combinatorial problem that allowed obtaining the best pair of $\eta_{\mathrm{FOD}}$ and IFSS values. Figure 6a,b, show high accuracy between the experimental and theoretical results. The estimated values of IFSS, the measured values of $\left(\eta_{\mathrm{FOD}}\right)$ and the residual standard deviation $\left(\hat{S}\right)$ of the simulated stress-strain curves are summarized in Table 3. The model was compared with an average curve representative of the experiments carried out for the two configurations of the compound. We have used the offset method at 0.002 mm/mm and the differences are not as marked as you point out. In the first case Figure 6a, the model underestimates the yield stress by approximately 15% and in the second case Figure 6b, in larger contents it overestimates it by 3%. It can be seen in the figures shown below. However, it is worth noticing that there are two important variations between the simulations and the experimental results. The first variation corresponds to a tendency to overestimate the stress when the deformation increases. This effect is more important when the fiber content is increased (e.g., 40 wt%.), because the fraction of subcritical fibers is greater (Figure 4a); therefore, the model will overestimate its contribution (residual standard error 1.42 MPa). This effect is due to the pull out of the subcritical fibers when the deformation is increased. The second variation is the inflection (softening) of the experimental curves which is not adequately reproduced in the simulations. This effect is caused by a phenomenon of ductile matrix damage which is not contemplated in the present model.

The present model presents substantial advantages with respect to the classic discretized model of Kelly–Tyson and its solution proposed by Bowyer and Bader, because it estimates the micromechanical parameters from the whole experimental stress-strain curve rather than two points arbitrarily selected. This method also allows to determine the accuracy of the predictions by means of the residual standard deviation. Nciri et al. [50] modeled the tensile behavior of PP+GF composite including fiber-matrix interfacial damage and ductile damage of the matrix, reducing the difference between experimental results and simulations; nevertheless, it required a complex phase of characterization of micromechanical parameters that limits its application for practical purposes. In the constitutive model, Notta-Cuvier et al. [27] the progressive separation of flax fibers and reduced the stress overestimation leading to accurate fit. Unfortunately, none of the previous models report the relative error or the residual standard deviation of the simulations.

Despite the mentioned limitations, the model accurately predicts the tensile behavior of the developed composite materials. In Table 4, a comparison is made between the results obtained through simulations and the available literature. The values highlighted in bold correspond to composite using MAPP as a coupling agent.

#### 4.3.1. Interface Shear Stress (IFSS)

The value of the interface shear strength (τ) was 12 MPa for the two compositions analyzed. This result is in the range of the values reported by the literature for polypropylene composite reinforced with lignocellulosic fibers and compatibilized with MAPP (bold values Table 4). In addition, this value is very close to the limit values proposed by the Von Mises criteria $\left(SS_{Mises}=14.9\;\mathrm{MPa}\right)$ and Tresca $\left(SS_{Tresca}=12.9\;\mathrm{MPa}\right)$ [18,49,55,56].

#### 4.3.2. Orientation Factor Efficiency ($\eta_{FOD}$)

The average value of the orientation efficiency factor for the two compositions (PP+30GAK_S_+4M and PP+40GAK_S_+8M) was 0.30. This value corresponds to an orientation of 42º. The angle is similar to that obtained by Serrano et al. [18] with an average angle of 39.9º for natural sisal fibers, as reported by López et al. [56] with an average angle of 43º for wood pulp (SGW) and to that reported by Vallejos et al. [55] with angle of 43º for fibers of hemp. The orientation efficiency factor was slightly higher in the case of composite with the following configuration PP+40GAK_S_, this effect is explained by the higher fiber content that increases the viscosity of the composite and reduces the mobility of the fiber bundles in its interior.

#### 4.3.3. Critical Length to Breakage Deformation ($l_{c}$)

The critical length at the breakage deformation was determined by the simulations and verified by Equation (6). The predicted values are within the values reported by the literature for PP composite reinforced with lignocellulosic fibers and compatibilized with MAPP (bold values Table 4). However, it is important to note that the critical length is a function of the deformation of the composite so that an evaluation of its effect will be made in the following section.

#### 4.3.4. Contribution of the Matrix and Fibers to the Tensile Strength of the Composite Materials

**Figure 7:**
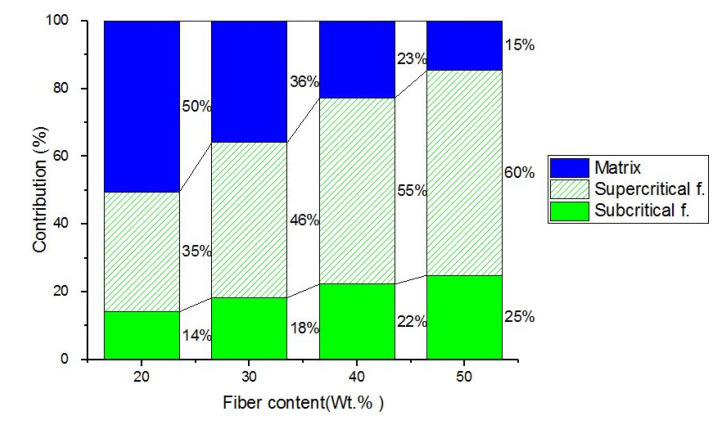
Contribution of subcritical, supercritical, and matrix fibers on the tensile strength of PP+GAKs+M composite materials.

Figure 7 shows the contributions of the subcritical fibers, supercritical, and matrix to the tensile strength of the composite materials obtained through the proposed model. Simulations were made from the adjusted model (In-Built) for fiber contents of 20, 30, 40 and 50 wt%. The contribution of subcritical fibers was lower, but it became more important as the fiber content increased. In the case of the PP+20GAK_S_ composite represents 14% of the total composite and reached up to 25% in the PP+50GAK_S_ composite (simulated). The contributions of the supercritical fibers represented 35%, 46%, 55%, and 60%, respectively. If the contributions of the fibers are accumulated, contributions of 49%, 64%, 77%, and 85% respectively are reached. This behavior evidences the effects of both content and length of the fiber bundles of GAK_S_ on the tensile strength of the formulated composite.

#### 4.3.5. Variation of Fiber Length and Orientation Efficiencies with Deformation

The implemented model allows us to evaluate the efficiency of the fiber length as the composite is deformed. At very small strains, most fibers are highly efficient at supporting the stresses transferred by the matrix. As the deformation increases, the efficiency is lost in the case of subcritical fibers. From Equation (10), it is possible to obtain the efficiency factor of length as a function of the deformation of the composite. What is interesting is that a non-linear decrease is observed until the material breaks.

**Figure 8:**
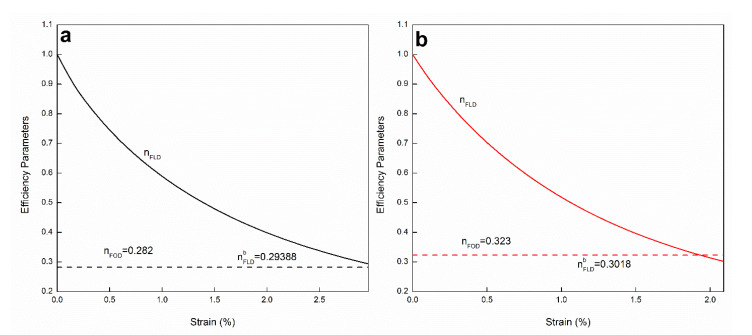
Variation of the factor of efficiency of length with the deformation of the composite PP+GAKS: (**a**) PP+30GAKS and (**b**) PP+40GAKS.

In Figure 8a,b, the evolution of orientation efficiency and fiber length factors is observed. As the hypotheses of the model suggest, the $\eta_{\mathrm{FOD}}$ is independent of the deformation (see broken lines). On the contrary, the dependence of the length factor $\eta_{\mathrm{FLD}}$ with the deformation shows a non-linear loss of the reinforcing effect of up to 70% in the two composites, as the deformation increases, until reaching the break (solid lines). This continuous loss of $\eta_{\mathrm{FLD}}$ accompanied by the non-linear viscoelastic behavior of the PP thermoplastic matrix explains the non-linear tensile behavior of the formulated composite materials (Figure 2).

Each curve starts from a value of 1, which suggests that all fibers are greater than the critical length at strain value close to 0. As the strain increases, the critical length increases linearly, according to Equation (2) and the $\eta_{\mathrm{FLD}}$ decreases non-linearly according to Equation (10) until reaching the breaking strain of the composite $\left(\varepsilon_{b}\right).$

## 5. Conclusions

The prediction of the tensile properties of SNFCs is of great importance for the design of products with this type of material, generally obtained by injection molding. In this paper, an integral model (In-Built) was adjusted to predict the entire stress-strain curve of a polypropylene composite material reinforced with short natural fibers of *Guadua angustifolia*. The model was fed with real data on the orientation state of the fibers and the length distribution obtained by X-ray microtomography and data processing. A methodology was validated based on an integral In-Built model for estimating the tensile properties of a PP+GAK_S_+MAPP composite material to different compositions. It started with a process of characterization of the intrinsic properties of its constituents and the micromechanical parameters that influence the tensile behavior of the composite material. The specimens of the injected PP+GAK_S_+MAPP composite material were characterized by X-ray microtomography, to evaluate the distribution of lengths, orientations, and aspect ratio of the fibers inside the composite. It was determined that there is a reduction in the dimensions of the fiber bundles during extrusion + injection processing, which implies changes in the micromechanical parameters. It was determined that at higher fiber contents its length is reduced. A function of length distribution was obtained for the different fiber contents represented by the scale (K) and shape (n) parameters. The current proposed model presents substantial advantages over the classic discretized Kelly–Tyson model and its solution proposed by Bowyer and Bader, since it can reproduce the entire stress-strain curve of natural composite materials at 30 and 40 wt% reinforcement. The estimated values of the shear strength at the interface (τ) are not affected by the fiber content. This micromechanical parameter depends on the interface treatment. The values estimated using the In-Built method are more conservative than the theoretical limits proposed by Tresca and Von Misses. With the proposed model it was possible to estimate the contributions of the sub and supercritical fibers on the tensile stress. The dependence of the length factor $\eta_{\mathrm{FLD}}$ was identified with the strain. The loss of the reinforcing effect reached 70% upon reaching the ultimate strain. This continuous loss of $\eta_{\mathrm{FLD}}$, accompanied by the non-linear viscoelastic behavior of the PP thermoplastic matrix, explains the non-linear behavior of the formulated composite materials.

## Figures and Tables

**Table 1:** Mean values and standard deviations (SD, in parentheses) of the tensile mechanical properties of PP+GAKs+MAPP composites and raw PP.

Composite Code	Tensile Strength (MPa)	Tensile Yield Stress ^b^ (MPa)	Young’s Modulus (GPa)	Yield Strain (%)	Breaking Strain (%)
PP+30GAKs+4M	35.79 (1.4)	27.6 (0.2)	3.44 (0.1)	1(0.1)	3 (0.1)
PP+30GAKs+8M	36.40 (1.2)	28.8 (0.2)	3.49 (0.1)	1 (0.1)	3 (0.1)
PP+40GAKs+4M	39.27 (1.1)	34.3 (0.3)	4.11 (0.1)	1 (0.1)	2 (0.1)
PP+40GAKs+8M	39.10 (0.3)	34.5 (0.3)	4.20 (0.2)	1 (0.1)	2 (0.1)
PP	25.82 (0.6) ^a^	16.54 (0.3)	1.21 (0.2)	9 (0.3)	--

^a^ Tensile stress (Yield). ^b^ At 0.2% offset.

**Table 2:** Input parameters for modeling tensile behavior of PP+GAKs composites.

Parameter	Unit	PP+30GAK_s_	PP+40GAK_s_
Scale (K)		0.001659	0.0009827
Shape (n)		1.326	1.571
$\mathrm{Fiber}\;\mathrm{modulus}\;(E_{f}$)	(MPa)	21,130	21,130
$\mathrm{Matrix}\;\mathrm{modulus}\;\left(E_{m}\right)$	(MPa)	1210	1210
Ultimate strain composite (ε)	(%)	3.00	2.00
Fiber diameter (d)	(μm)	28.57	21.01
$\mathrm{Volume}\;\mathrm{fraction}\;\left(V_{f}\right)$		0.27	0.36

**Table 3:** Micromechanical output parameters τ and $\eta_{FOD}$ of the model (In-Built). $\hat{S}$ is the residual standard deviation of the estimated stress-strain curve.

Composition	τ (MPa)	$\eta_{FOD}$	$\hat{S}\;(\mathrm{MPa})$
PP+30GAKs	12	0.282	0.78
PP+40GAKs	12	0.323	1.42

**Table 4:** Micromechanical parameters reported by the literature for traction modeling of PP+lignocellulosic fiber composite.

Author	Matrix	Reinforcement (wt%)	τ (MPa)	$\eta_{FOD}$	$l_{C}\;\left(\mu m\right)$
Modniks [59]	PP	Flax 20	4–8	N/D	N/D
Notta Cuvier [27]	PP	Flax 30	3.78	N/D	N/D
Serrano [18]	PP+MAPP	ONPs 20	14.5	0.37	N/D
		ONPs 50	13.91	0.32	
Serrano [23]	PP	ONPs 20	N/D	0.49	N/D
		ONPs 50		0.57	
López [56]	PP	SGW 30	3.85	0.37	1277
		SGW 50	3.45	0.36	1342
	PP+MAPP	SGW 30	15.71	0.29	649
		SGW 50	15.87	0.28	566
Vallejos [55]	PP+MAPP	Hemp 20	14.95	0.28	N/D
		Hemp 50	15.6	0.28	
Li [60]	PP	Hemp 40	6.69	0.44	N/D
Fajardo ^a^	PP+MAPP	GAKs 30	12	0.282	536
		GAKs 40	12	0.323	394

^a^ Data of the present study.

## Data Availability

Data is contained within the article.
